# A stable pattern of EEG spectral coherence distinguishes children with autism from neuro-typical controls - a large case control study

**DOI:** 10.1186/1741-7015-10-64

**Published:** 2012-06-26

**Authors:** Frank H Duffy, Heidelise Als

**Affiliations:** 1 Department of Neurology, Children's Hospital Boston and Harvard Medical School, 300 Longwood Ave., Boston, MA 02115, USA; 2 Department of Psychiatry(Psychology), Children's Hospital Boston and Harvard Medical School, 320 Longwood Ave., Boston, MA 02115, USA

**Keywords:** Autism spectrum disorder, pervasive developmental disorder, PDD, EEG coherence, principal components analysis, PCA, coherence factors, discriminant analysis

## Abstract

**Background:**

The autism rate has recently increased to 1 in 100 children. Genetic studies demonstrate poorly understood complexity. Environmental factors apparently also play a role. Magnetic resonance imaging (MRI) studies demonstrate increased brain sizes and altered connectivity. Electroencephalogram (EEG) coherence studies confirm connectivity changes. However, genetic-, MRI- and/or EEG-based diagnostic tests are not yet available. The varied study results likely reflect methodological and population differences, small samples and, for EEG, lack of attention to group-specific artifact.

**Methods:**

Of the 1,304 subjects who participated in this study, with ages ranging from 1 to 18 years old and assessed with comparable EEG studies, 463 children were diagnosed with autism spectrum disorder (ASD); 571 children were neuro-typical controls (C). After artifact management, principal components analysis (PCA) identified EEG spectral coherence factors with corresponding loading patterns. The 2- to 12-year-old subsample consisted of 430 ASD- and 554 C-group subjects (n = 984). Discriminant function analysis (DFA) determined the spectral coherence factors' discrimination success for the two groups. Loading patterns on the DFA-selected coherence factors described ASD-specific coherence differences when compared to controls.

**Results:**

Total sample PCA of coherence data identified 40 factors which explained 50.8% of the total population variance. For the 2- to 12-year-olds, the 40 factors showed highly significant group differences (*P *< 0.0001). Ten randomly generated split half replications demonstrated high-average classification success (C, 88.5%; ASD, 86.0%). Still higher success was obtained in the more restricted age sub-samples using the jackknifing technique: 2- to 4-year-olds (C, 90.6%; ASD, 98.1%); 4- to 6-year-olds (C, 90.9%; ASD 99.1%); and 6- to 12-year-olds (C, 98.7%; ASD, 93.9%). Coherence loadings demonstrated reduced short-distance and reduced, as well as increased, long-distance coherences for the ASD-groups, when compared to the controls. Average spectral loading per factor was wide (10.1 Hz).

**Conclusions:**

Classification success suggests a stable coherence loading pattern that differentiates ASD- from C-group subjects. This might constitute an EEG coherence-based phenotype of childhood autism. The predominantly reduced short-distance coherences may indicate poor local network function. The increased long-distance coherences may represent compensatory processes or reduced neural pruning. The wide average spectral range of factor loadings may suggest over-damped neural networks.

## Background

Autism, also referred to as autism spectrum disorder (ASD), constitutes a neurodevelopmental disorder characterized by impairment in communication, including language, social skills and comportment often involving rigidity of interests and repetitive, stereotypical behaviors [1]. Ancillary symptoms may encompass obsessive-compulsive, sleep, hyperactivity, attention, mood, gastrointestinal, self-injurious, ritualistic, and sensory integration disorders. ASD is generally considered a life-long disability of yet undetermined etiology, without an established confirmatory laboratory test, and as yet without universally established, curative pharmacological or behavioral therapy [2-4]. The incidence of autism appears to be increasing. In 2011, Manning *et al. *[5] using birth certificate and Early Intervention data reported that in the Commonwealth of Massachusetts between 2001 and 2005 the incidence of ASD diagnosed by 36 months of age increased from 56 to 93 infants per 10,000. Whether this increased incidence reflects better reporting and/or diagnosis or whether other factors are involved remains to be determined. None-the-less, such an increase in incidence is alarming. These data appropriately have spawned much research into the exploration of potential etiologies as well as the development of diagnostic tests, particularly in terms of neuro-imaging and EEG, with the hope of establishing a definitive diagnosis at the earliest possible age, in order to facilitate early intervention, while the immature brain still holds high compensatory promise.

ASD is considered by many to be a genetically determined disorder; three well-known twin studies [6-8] estimate heritability at about 90% [9]. Sibling concordance varies from about 3 to 14%; linkage studies are consistent with a polygenic mode of transmission [10]. The 2008 finding by the Autism Consortium of a microduplication at 16p11.2 (1% of studied cases) raised hopes that a full ASD genomic pattern might soon be elucidated. However, more recent data suggest the heterogeneity and complexity of genetic abnormalities identified in children with ASD. Sakai *et al. *[11] set out with 26 ASD associated genes and then described an "interactome" of autism-associated proteins that may be necessary to describe common mechanisms underlying ASD. Voineagu *et al. *[12] provided strong evidence to suggest widespread transcriptional and splicing dysregulation as the key mechanism underlying brain dysfunction in ASD. On the basis of a detailed study of twins with autism, Hallmayer *et al. *[9] recently reported, as expected, high twin concordance yet also concluded that ASD has, in addition to moderate heritability, a substantial environmental component. Thus, studies to date suggest a strong genetic component to autism that may, however, be more complex than initially thought, and environmental factors, especially their types and mechanisms of action, also appear to deserve further consideration.

MRI and its derivatives have demonstrated important findings in ASD as has been reviewed extensively [13-16]. The earliest anatomical studies involved recognition that young children with ASD have abnormally increased total brain volumes that appear related to both increased grey and white matter volumes, with a differentially higher white matter contribution. Brain size in ASD appears to reach a 10% increase beyond control values by two to four years of age, possibly followed by a plateau. Regional brain growth specificity studies, however, have shown little consistency with the exception of decreased corpus callosum volume in ASD suggesting decreased interhemispheric connectivity. Diffusion magnetic resonance imaging (DMRI) studies in children and adults have demonstrated lower white matter tract fractional anisotropy (FA) in ASD, indicating poorer functional connectivity between brain regions. Supporting this, Just *et al. *[17,18] published functional MRI (fMRI) studies which demonstrate functional under-connectivity in ASD. However, some studies have provided evidence for several regions with increased FA, that is, likely increased connectivity, in both children and adolescents with ASD [19,20].

As Chen [16] correctly pointed out, there are "many conflicting... (MRI)... findings in individuals within the ASD...(which result from)...factors such as population age, MRI acquisition parameters, details of the image processing pipeline, feature extraction procedures, analytic methods used to detect group differences and sample sizes...(which have)...contributed to these disparities...". From the entirety of MRI related studies, one may conclude that ASD is typically associated with widely distributed alterations of brain anatomy involving both grey and white matter, and with alterations in functional connectivity, which appear primarily decreased, yet also with some regionally increased connectivity. Despite a number of serious attempts, there are as yet no universally established MRI-based criteria that are usable to diagnose ASD. This no doubt reflects the problematic complexity of factors underlying autism as outlined above.

Given that altered brain connectivity is considered a typical characteristic of ASD, a number of studies have compared EEG coherence findings between ASD and neuro-typical control populations [21-28]. On a frequency by frequency basis, EEG spectral coherence represents the consistency of the phase difference between two EEG signals when compared over time. According to Srinvasan *et al. *"...coherence is a measure of synchronization between two... (EEG)...signals based mainly on phase consistency; that is, two signals may have different phases... but high coherence occurs when this phase difference tends to remain constant. In each frequency band, coherence measures whether two signals can be related by a linear time invariant transformation, in other words a constant amplitude ratio and phase shift (delay). In practice, EEG coherence depends mostly on the consistency of phase differences between channels" [29]. High coherence values are taken as a measure of strong connectivity between the brain regions that produce the compared EEG signals [30].

There is general agreement among coherence study results that ASD patients and neuro-typical subjects differ markedly in terms of coherence findings; however, as for MRI, study details also differ markedly. Cantor *et al. *[21], who studied a small group of 4- to -12-year-old children with ASD, reported greater between-hemisphere coherence in the children with autism than in comparable age children with mental handicaps other than autism. Murias *et al. *[22] evaluated 18 adults with ASD and found locally elevated theta coherence, especially in the left hemisphere. Alpha coherence was reduced within the frontal and between the frontal and other regions. Coben *et al. *[23] studied 20 6- to 11-year-old children with ASD and reported decreased overall coherence compared to neuro-typical control group children. The children with ASD demonstrated decreased intrahemispheric delta and theta for both short and long inter-electrode distances as well as similarly decreased interhemispheric coherence. Lazarev *et al. *[24] evaluated, with EEG during photic stimulation at different frequencies, 14 6- to 14-year-old children with ASD in comparison to a neuro-typical control group. The authors reported an ASD-specific coherence increase at the frequencies of stimulation in the left but not the right hemisphere, as compared to the neuro-typical subjects. Resting, that is, not specifically stimulated, coherence did not differ between the two hemispheres for either group. Isler *et al. *[25] evaluated coherence between two homologous regions of visual cortex during visual stimulation (long latency evoked potentials) in nine children with ASD as compared to neuro-typical controls. The children with ASD demonstrated significantly reduced coherence in the delta and theta spectral bands and essentially no interhemispheric synchronization above the theta band, whereas the neuro-typical children sustained interhemispheric synchrony to higher frequencies. This suggested diminished functional connectivity between the bihemispheric visual regions during visual stimulation in ASD. Leveille *et al. *[27] assessed resting EEG coherence during REM sleep in nine subjects with ASD compared to neuro-typical controls and reported greater coherence between the left occipital area and both local and distant regions for the children with ASD. They also reported lower coherence over right frontal regions for the children with ASD as compared to the control group. Sheikhani *et al. *[26] reported bilaterally increased coherence in the gamma band, especially involving the temporal lobes, in 17 subjects with ASD, ranging in age from 6 to 11 years, when compared to a healthy control group. Barttfeld *et al. *[28] evaluated 10 adults with ASD and noted that the subjects demonstrated reduced long-distance and also increased short- distance coherence when compared to an adult control group.

Study differences in experimental design, including choice of spectral bands, brain regions, brain states (activated or resting) and type of analysis, as well as small sample sizes, differences in sample age ranges, diversity of severity of impairment, lack of replication tests and disparity of results make difficult a meaningful summary of spectral coherence findings in ASD. Furthermore, few studies considered the reality of ASD group-specific EEG artifacts, including eye blink and muscle movement, and their potential spurious effects upon coherence. Also, few studies addressed the confounding effect of differing EEG recording reference techniques upon coherence [31]. This leaves wide open the question of whether the reported diverse study findings reflect marked variability of brain function within the ASD population as suggested by Happé [32] and recently demonstrated by Milne [33], or whether they primarily reflect methodological variability.

The current study attempts to answer the as yet open question of coherence differences between children with ASD and neuro-typical healthy controls. To this end, EEG coherence data were evaluated in a large sample of children with ASD and compared to a large neuro-typical, medically healthy, normal, age-comparable control group. Care was taken to minimize the effects of EEG artifact upon coherence data and to avoid *a priori *selection of coherences from among the very large number of created coherence variables.

## Methods

### Study population

The Developmental Neurophysiology Laboratory, under the direction of the first author, maintains a database of patients and research subjects that includes unprocessed (raw) EEG data in addition to referral information. Patients typically are referred in order to rule out epilepsy and/or sensory processing abnormalities by studies incorporating EEG and Evoked Potentials (EP).

#### Patients with ASD

The goal of the current study was to select only those patients whom experienced clinicians recognized and identified as patients on the autistic spectrum, while excluding children in the extremes of this entity, confounding neurological diagnoses that may present with autistic features, and other entities that might have independent impact upon EEG data.

Necessary inclusion criteria included the diagnosis of ASD or Pervasive Developmental Disorder not otherwise specified (PDD-nos) - both hereafter bundled and together referred to as ASD - as determined by an independent pediatric neurologist, psychiatrist, or psychologist at CHB or at one of several other Harvard teaching hospitals, specializing in childhood developmental disabilities, including ASD. Diagnoses relied upon DSM-IV [1] and/or ADOS [34-36] criteria aided by clinical history and expert team evaluation.

Exclusion criteria included: (1) co-existing primary neurologic syndromes that may present with autistic features (for example, Rett's, Angelman's and Fragile X syndromes, tuberous sclerosis, or mitochondrial disorders); (2) clinical seizure disorders or results of EEG readings suggestive of an active seizure disorder or epileptic encephalopathy. (Note: Patients with occasional EEG spikes were not excluded); (3) a primary diagnosis of global developmental delay (GDD), developmental dysphasia or high functioning autism and/or Asperger's syndrome; (4) expressed doubt by the referring clinician as to the diagnosis of ASD; (5) taking medication(s) at the time of the study; (6) other concurrent neurological disease processes that might induce EEG alteration, for example, hydrocephalus, hemiparesis or known syndromes affecting brain development; and (7) significant primary sensory disorders, for example, blindness and/or deafness. A total of 463 patients met the above study criteria and were designated as the study's ASD sample.

#### Healthy controls

From among normal children recruited and studied for developmental research projects, the goal was to provide a comparison group of children selected to be normally functioning while avoiding creation of an exclusively 'super-normal' group. For example, subjects with the sole history of prematurity or low-weight birth, and not requiring medical treatment after birth hospital (Harvard affiliated hospital) discharge were included.

Necessary inclusion criteria were as follows: (1) living at home with and considered normal by the parents; and (2) identified as functioning within the normal range on standardized developmental and/or neuropsychological assessments performed during the respective research study.

Exclusion criteria were as follows: (1) diagnosed neurologic or psychiatric illness or disorder or expressed suspicion of such, for example, global developmental delay (GDD), developmental dysphasia, attention deficit disorder (ADD) and attention deficit with hyperactivity disorder (ADHD); (2) abnormal neurological examination as identified during the research study; (3) clinical seizure disorder or EEG reading suggesting an active seizure disorder or epileptic encephalopathy (Note: Subjects with rare EEG spikes were not excluded); (4) noted by the research psychologist or neurologist to present with autistic features; (5) newborn period diagnosis of intraventricular hemorrhage (IVH), retinopathy of prematurity, hydrocephalus, or cerebral palsy or other significant condition likely influencing EEG data; and/or (6) taking medication(s) at time of EEG study. A total of 571 patients met the criteria for neuro-typical controls and were designated as the study's control (C) sample.

#### Institutional Review Board approvals

All control subject families, and subjects as age appropriate, gave informed consent in accordance with protocols approved by the Institutional Review Board (IRB) of Children's Hospital Boston. Subjects with ASD who had been referred clinically were studied under an IRB protocol that solely required de-identification of data without requirement of informed consent.

### Measurements and data analysis

#### EEG data acquisition

**Figure 1:**
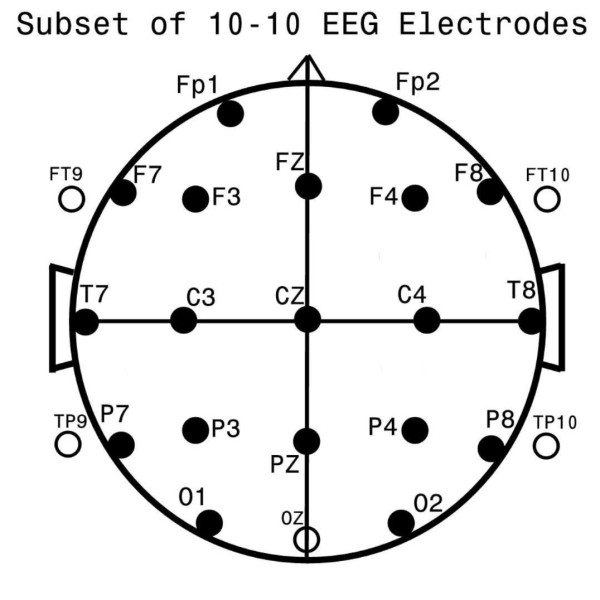
**Standard EEG electrode names and positions**. Head in vertex view, nose above, left ear to left. EEG electrodes, Z, Midline, FZ, Midline Frontal; CZ, Midline Central; PZ, Midline Parietal; OZ, Midline Occipital. Even numbers, right hemisphere locations; odd numbers, left hemisphere locations, Fp, Frontopolar; F, Frontal; C, Central; T, Temporal; P, Parietal; O, Occipital. The standard 19, 10 to 20 electrodes are shown as black circles. An additional subset of five, 10-10 electrodes are shown as open circles.

Registered EEG technologists, naïve to the study's goals, and specifically trained and skilled in working with children within the study's age group and diagnostic range, obtained all EEG data by use of up to 32 gold-cup scalp electrodes applied with collodion after measurement. Analyses were subsequently restricted to the following 24 channels available for all subjects: FP1, FP2, F7, F3, FZ, F4, F8, T7, C3, CZ, C4, T8, P7, P3, PZ, P4, P8, O1, OZ, O2, FT9, FT10, TP9, TP10 (see Figure 1). EEG data were gathered in the awake and alert state assuring that adequate periods of waking EEG were gathered. EEG data collected during EP formation were not utilized for the study. Data were primarily obtained from Grass™ (Grass Technologies Astro-Med, Industrial Park 600, East Greenwich Avenue, West Warwick, RI 02893 USA) EEG amplifiers with 1 to 100 Hz bandpass filtering and digitized at 256 Hz for subsequent analyses. All amplifiers were individually calibrated prior to each study. One other amplifier type was utilized for five patients with ASD (Bio-logic™, Bio-logic Technologies, Natus Medical Inc., 1501 Industrial Road, San Carlos, CA 04070 USA; 250 Hz sampling rate, 1 to 100 Hz bandpass) and one other amplifier type was utilized for 11 control subjects (Neuroscan™, Compumedics Neuroscan, 6605 West W.T. Harris Boulevard, Suite F, Charlotte, NC 28269 USA, 500 Hz sampling rate, 0.1 to 100 Hz bandpass). Data from these two amplifiers, sampled at other than 256 Hz. were interpolated to the rate of 256 Hz by the BESA 3.5™ software package. As the band-pass filter characteristics differed among the three EEG machines, frequency response sweeps were performed on all amplifier types so as to permit modification of data recorded with the Biologic and Neuroscan amplifiers to be equivalent to those gathered by the Grass amplifiers. This was accomplished by utilizing special software developed in-house by the first author using forward and reverse Fourier transforms [37].

#### Measurement issues and solutions

EEG studies are confronted with two major methodological problems. First is the management of the abundant artifacts observed in young and behaviorally difficult to manage children (for example, eye movement, eye blink and muscle activity). It has been well established that even EEGs appearing clean by visual inspection may contain significant artifacts [38,39]. Moreover, as shown in schizophrenia EEG research, certain artifacts may be group specific [40]. Second is the capitalization upon chance, that is, application of statistical tests to too many variables and incorrect reports of those that appear significant by chance as support for the experimental hypothesis [41]. Methods discussed below were designed to specifically address these problems.

##### Artifact management - Part 1: Unprocessed EEG signals

At the conclusion of each subject's data collection, digitized EEG data were inspected by the EEG technologist and those EEG epochs were visually identified which were recorded during breaks for relaxation, or showed movement artifact, electrode artifact, eye blink storms, drowsiness, epileptiform discharges, and/or bursts of muscle activity. Once identified, they were marked in order to allow complete exclusion from subsequent analyses of all channels recorded during such epochs. Results were reviewed and confirmed and/or modified by an experienced pediatric electroencephalographer (first author). After such visual inspection and treatment, data were low pass filtered below 50 Hz with an additional 60 Hz mains rejection notch filter. Remaining eye blink and eye movement artifacts, which may be surprisingly prominent even during the eyes closed state, were removed by utilizing the source component technique [42,43] as implemented in the BESA (BESA GmbH, Freihamer Strasse 18, 82116 Gräfelfing - Germany) software package. These combined techniques resulted in EEG data that appeared largely artifact free, with rare exceptions of low level temporal muscle artifact and persisting frontal and anterior temporal slow eye movement, which remain capable of contaminating subsequent analyses. The final reduction of such persisting contamination of processed variables (coherence) is discussed below under *Artifact management - Part 2*

##### Calculation of spectral coherence variables

Approximately 8 to 20 minutes of awake state EEG data per subject were transformed by use of BESA software, which supplies an implementation of a spherical spline algorithm [44] to compute scalp Laplacian or current source density (CSD) estimates for surface EEG studies. The CSD technique was employed as it provides reference independent data that are primarily sensitive to underlying cortex and relatively insensitive to deep/remote EEG sources. Srinvasan *et al. *[29] point out that..."EEG coherence is often used to assess functional connectivity in human cortex. However, moderate to large EEG coherence can also arise simply by the volume conduction of current through the tissues of the head... (and)...EEG coherence appears to result from a mixture of volume conduction effects and genuine source coherence. Surface Laplacian EEG methods minimize the effect of volume conduction on coherence estimates by emphasizing sources at smaller spatial scales than unprocessed potentials (EEG)."

Spectral coherence was calculated, using a Nicolet™ (Nicolet Biomedical Inc., 5225 Verona Road, Madison, WI 53711 USA) software package, according to the conventions recommended by van Drongelen [30] (pages 143-144, equations 8.40, 8.44). Coherency [45] is the ratio of the cross-spectrum to the square-root of the product of the two auto-spectra and is a complex-valued quantity. Coherence is the square modulus of coherency, taking on a value between 0 and 1. In practice, coherence is typically estimated by averaging over several epochs or frequency bands [30] and in the current project a series of two second epochs were utilized over the total available EEG segments.

Furthermore, the quest for better measures of connectivity between brain regions in EEG and MRI has recently generated new techniques for connectivity assessment in MRI and EEG [46-48]. Such techniques involve partial coherence as the measure of functional connectivity and appear particularly useful when comparing connectivity across tasks. As this was not the case in the current study, partial coherence was not utilized for the current project.

Spectral coherence measures were derived from the 1 to 32 Hz range, in 16, two-Hz-wide, spectral bands which results in 4,416 unique coherence variables. The 24 by 24 electrode coherence matrix yields 576 possible coherence values; the matrix diagonal has a value of 1 - each electrode to itself - and half of the 552 remaining values duplicate the other half, which results in 276 unique coherences per spectral band. Multiplication by the 16 spectral bands in turn results in 4,416 unique spectral coherence values per subject.

##### Artifact management - Part 2: Coherence data

As has been recently discussed in a study of normal adults and adults with chronic fatigue syndrome [49], artifacts cannot be removed from an entire EEG data set alone by visual inspection and direct elimination of electrodes and/or frequencies where a particular artifact is most easily apparent. An established approach to reduce further any persisting artifact contamination of processed coherence data involves multivariate regression. Semlitsch *et al. *[50] demonstrated that by identifying a signal that is proportional to a known source of artifact, this signal's contribution to scalp recorded data (EEG and its derivatives, such as evoked potentials, and so on) may be diminished by statistical regression procedures. Persisting vertical eye movements and blinks produce slow EEG delta spectral signals in the frontopolar channels FP1 and FP2 and such artifactual contribution may be estimated by the average of the 0.5 and 1.0 Hz spectral components from these channels after EEG spectral analysis by Fast Fourier Transform (FFT) [37] of common average referenced data. Similarly, horizontal eye movements may be estimated by the average of the 0.5 to 1.0 Hz spectral components from the anterior temporal electrodes F7 and F8. Little meaningful information of brain origin is typically found at this slow frequency in these channels in the absence of extreme pathology. Muscle activity tends to peak at frequencies above those of current interest. Accordingly, 30 to 32 Hz spectral components were considered to be largely representative of muscle contamination, especially as recorded from the separate averages of prefrontal (FP1, FP2), anterior temporal (F7, F8), mid-temporal (T7, T8), and posterior temporal (P7, P8) electrodes. These electrodes are the ones most often contaminated by muscle as they are physically closest to the source of the artifact (frontal and temporal muscles). The steps employed in this study involved, first, the fitting of a linear regression model where the dependent variables were those targeted for artifact reduction and the independent variables were those chosen as representative of remaining artifacts; second, the extracting of the residuals which now represent the targeted data with artifacts removed and, third, the use of these residuals in subsequent analyses. The six artifact measures, two very slow delta and four high frequency beta, were the ones submitted as independent variables to the multiple regression analysis (BMDP2007™-6R) [51], which was used to individually predict each of the coherence variables (see below), treated as dependent variables. Residuals of the dependent variables, now uncorrelated with the chosen independent artifact variables, were used in the subsequent analyses.

#### Prevention of capitalization upon chance: Variable number reduction by creation of coherence factors

In order to facilitate subsequent statistical analysis, specifically in order to avoid capitalization on chance resulting from the use of too many variables, Principal Components Analysis (PCA) of the coherence data was employed as an objective technique to meaningfully reduce variable number [52]. The coherence data were first normalized (centered and shifted to have unit variance) so that eventual factors reflected deviations from the average. In order to avoid loss of sensitivity by *a priori *data limitation, an unrestricted form of PCA [53] was applied allowing all coherence variables per subject to enter analysis. By employment of an algorithm based upon singular value decomposition (SVD) [37,54], a data set of uncorrelated (orthogonal) principal components or factors [52,53] was developed in which the identification of a small number of factors following Varimax rotation [55] describe an acceptably large amount of variance [56]. Varimax rotation enhances factor contrast yielding higher loadings for fewer factors while retaining factor orthogonality. Although not the only PCA method applicable to large, asymmetrical matrices (4,416 variables by 1,034 cases as in the current study), SVD, which may be used to solve under-determined and over-determined systems of linear equations [37], is among the most efficient techniques used for PCA [53]. This approach to variable number reduction has been successfully used in prior studies of EEG spectral coherence in infants [57] and adults [49,53]. When total population size is over 200, as in the current study, coherence factor formation consistency by split-half replication becomes redundant (unpublished finding).

#### Data analysis

##### Discrimination of subject groups by use of EEG spectral coherence variables

Two-group discriminant function analysis (DFA) [58-60] was used extensively in this study. It produced a new canonical variable, the discriminant function, which maximally separated the groups, based on a weighted combination of the entered variables. DFA defined the significance of a group separation, summarized the classification of each subject, and provided approaches to the prospective classification of subjects not involved in discriminant rule generation by means of the jackknifing technique [61,62] or by classification of a new population. The BMDP2007™ (Statistical Solutions, Stonehill Corporate Center, Suite 104, 999 Broadway, Saugus, MA 01906 USA) statistical package [51] was employed for DFA (program 7 M); it yields the Wilk's Lambda statistic with Rao's approximation. For the estimation of prospective classification success, the jackknifing technique was used [61,62]. In jackknifing for two-group DFA, as was undertaken in this study, the discriminant function was formed on all subjects but one. The left-out subject was subsequently classified. This initial left out subject was then folded back into the group (hence "jackknifing"), another subject was left out, the DFA was performed again, and the newly left out subject classified. This process was repeated until each individual subject had been left out and classified. The measure of classification success was then based upon a tally of the correct classifications of the left out subjects. This technique is also referred to as the "leaving-one-out" process. Split half analysis was also used. Instead of leaving out a single subject for each iteration, 50% of subjects were left out, that is, the analysis was performed on a randomly selected sample consisting of only half the number of subjects. A random number generator within BMDP-7M (stepwise DFA) was employed to permit random assignment of each subject to a training-set (50% of the subjects - used to create the discriminant) and a test-set (remaining 50% of the subjects - used to estimate prospective classification success). The algorithm used by BMDP does not always provide a precise split; the exact ratio of control to experimental subjects within each selected sub-group reflects random chance. As a separate measure of classification success, two-group t-tests (BMDP-3D) were performed utilizing the canonical discriminant variable produced by a training-set test on the corresponding test-set.

##### Factor description; relationship of PCA outcome factors to input coherence variables

**Figure 2:**
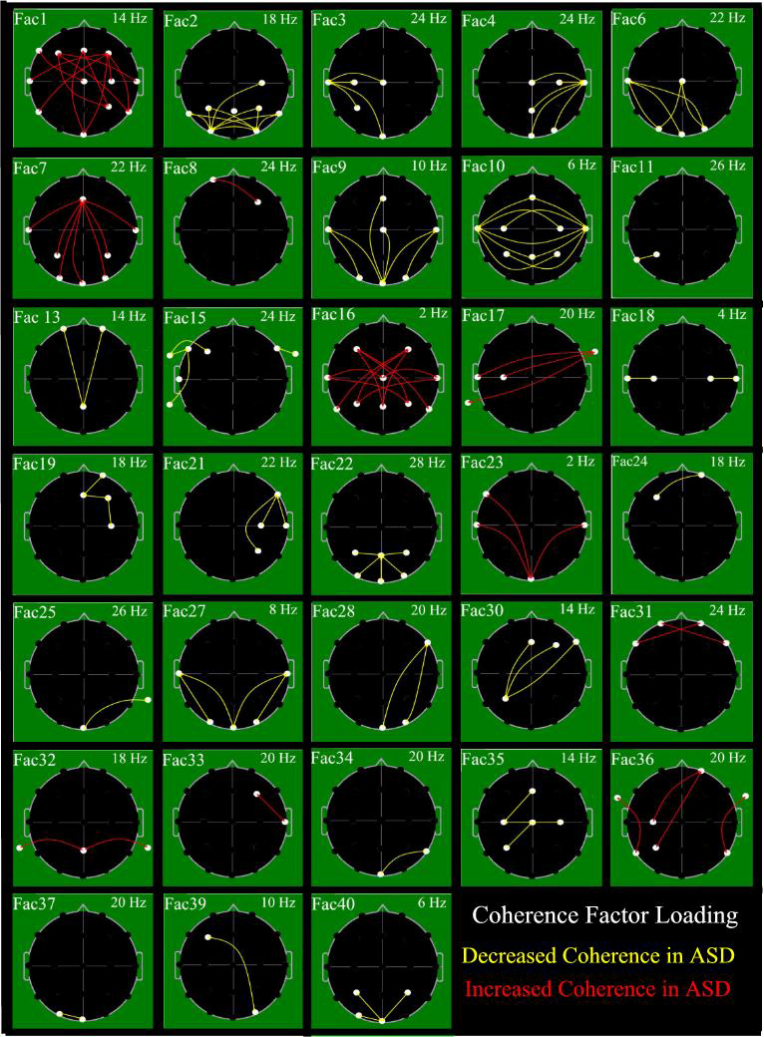
**Graphic representation of 33 coherence factor loadings**. EEG coherence factor loadings. Heads in top view, scalp left to image left, nose above; Factor number is above heads to left and peak frequency for factor in Hz is above to right. Lines indicate top 15% coherence loadings per factor: Red = increased coherence in ASD-group; Yellow = decreased coherence in ASD-group. Involved electrodes shown as small white circles. Uninvolved electrodes are not shown.

Individual outcome factors were each formed as linear combinations of all input variables with the weight or loading of each coherence variable upon a particular factor as determined by the PCA computation [58]. Meaning of outcome factors was discerned by inspection of the loadings of the input variables upon each individual factor [52,58]. Factor loadings were treated as if they were primary neurophysiologic data and displayed topographically [63,64]. Display of the highest 15% of coherence loading values, was utilized [49,53,57], to facilitate an understanding of individual factors' meaning, as shown in Figure 2.

#### Age grouping

Given the wide age range (14 months to 18 years) of the subjects within the ASD- and C-groups and the well known age effects on EEG and spectral coherence data over this wide age range [65-67], analyses were restricted to the more limited age range of 2 to 12 years (ASD-group: n = 430; C-group: n = 554; total sample: n = 984, see Table 1). A high male (84%) to female (16%) ratio in the ASD-group reflects known male preponderance for this population [68]. A similar pattern in the C-group (male (88%), female 12%) reflects intentional bias as subject selection anticipated studies of autism and other studies from which the C-group was drawn (for example, dyslexia, learning disabilities, and behavior problems where males predominate) [69,70]. Male to female ratios were not significantly different between the ASD- and C-groups. The effect of age was removed from the 40 coherence variables generated on the 2- to 12-year-old total sample by simple regression using age-at-study as the independent variable and the 40 coherence factors as dependent variables (BMDP-6R). Factors remained statistically uncorrelated after this regression procedure. In order to assure relatively even age distribution of subject numbers between ASD- and C-groups, group comparisons were also independently performed in three narrower age ranges, namely for 2- to 4-year-olds, 4- to 6-year-olds, and 6- to 12-year olds.

**Table 1:** Populations studied

Description	Total	Control	Autistic
Fulfilling Criteria, Used for PCA Ages 1 to 19 years	1,034	571	463
Used for Discriminant, Ages 2 to 12 years	984	554 16% female	430 12% female
Subgroup, 2 to 4 years	301	85	216
Subgroup, 4 to 6 years	137	22	115
Subgroup, 6 to 12 years	546	447	99

## Results

### Generation and selection of spectral coherence variables

Subsequent to SVD-based PCA, distribution of variation among coherence factors demonstrated a satisfactory condensation of variance into a small number of factors: 829 factors described over 99%, 366 described 90.02%, 38 described 49.98%, 7 described 24.97% and 1 factor described 8.10% of the total variance before rotation. The first 40 factors accounted for 50.87% of the total variance. Variance and percent variance after Varimax rotation are shown in Table 2. Factors were named in the order selected by their Eigenvalues before rotation. In Table 2, the percent variance values are not in descending order, which is an expected result of the variance re-distribution from the Varimax rotation. These 40 factors were used as variables to represent all subjects in the subsequent analyses.

**Table 2:** First 40 factors after varimax rotation

Factor Order	Variance	Percent of All Factors	Percent of First 40 Factors	Factor Order **Cont**.	Variance	Percent of All Factors	Percent of First 40 Factors
1	147.57	3.34	6.57	21	35.92	0.81	1.60
2	111.41	2.52	4.96	22	44.44	1.01	1.98
3	123.86	2.80	5.51	23	40.20	0.91	1.79
4	146.55	3.32	6.52	24	47.27	1.07	2.10
5	79.48	1.80	3.54	25	40.59	0.92	1.81
6	117.38	2.66	5.22	26	32.21	0.73	1.43
7	75.19	1.70	3.54	27	39.74	0.90	1.77
8	45.95	1.04	2.05	28	36.58	0.83	1.63
9	95.90	2.17	4.27	29	43.60	0.99	1.94
10	62.35	1.41	2.78	30	30.33	0.69	1.35
11	39.96	0.90	1.78	31	41.26	0.93	1.84
12	95.55	2.16	4.25	32	29.18	0.66	1.30
13	58.48	1.32	2.60	33	43.85	0.99	1.95
14	63.86	1.45	2.84	34	29.10	0.66	1.30
15	71.38	1.62	3.18	35	28.82	0.65	1.28
16	45.06	1.02	2.01	36	25.51	0.58	1.14
17	33.29	0.75	1.48	37	27.78	0.63	1.24
18	33.78	0.76	1.50	38	32.22	0.73	1.43
19	40.60	0.92	1.81	39	36.08	0.82	1.61
20	49.17	1.11	2.19	40	25.08	0.57	1.12

Total variance for all factors = 4,416.01

Total variance of first 40 Factors = 2,246.55 (50.87% of total variance)

*Factors are ordered and named on basis of variance before rotation.

### Analysis of entire 2- to 12-year-old sample analyses: two group DFA, 40 coherence factors

#### All 40 variables forced to enter

When the primary discriminant function analysis (DFA) was based upon the 2- to 12-year-old sample of 984 subjects and all 40 coherence factors were forced to enter the DFA, there was a significant group differentiation of the ASD- and C-groups by Wilks' Lambda (0.490) with Rao's approximation (F = 23.66; df = 40, 943; *P *< 0.0001). This result established that these two groups differed significantly on the basis of variables generated from EEG-based coherence data.

#### Split half replication with variable stepping

When DFA was performed with 10 replications, allowing variables to step in or step out each time after first randomly splitting the population into two parts, forming training- and test-sets. The average test-set classification success across all 10 split half replications was 88.5% for the C- and 86.0% for the ASD-group. Results are shown in Tables 3 and 4. The DFAs utilized between 19 and 25 factors; Factor 15 was chosen consistently as the first for each of the 10 replications (Table 3). When additionally confirmed by t-test all 10 scores reached significance at *P *≤ 0.0001 (Table 4). The consistent classification success and the highly significant t-test results for the 10 split half analyses indicate that stable, consistent differences exist between the C- and ASD-groups.

**Table 3:** Ten consecutive split-half replications of full population

Trial	Number of Training Set Subjects	Number of Test Set Subjects	Number of Factors Used	Top Two Factors Chosen
1	473	511	25	15, 1
2	469	515	20	15, 16
3	490	494	21	15, 16
4	521	463	21	15, 16
5	480	504	23	15, 16
6	487	497	25	15, 2
7	496	488	19	15, 17
8	490	494	22	15, 17
9	495	489	22	15, 17
10	501	483	22	15, 16

**Table 4:** Ten instances of split-half replication of full population

Trial	Num CON Correct	% CON Correct	Num ASD Correct	% ASD Correct	t	df	*P*
1	244/279	87.5	204/232	87.9	11.18	317	0.0001
2	256/297	86.2	195/218	89.4	12.95	304	0.0001
3	253/285	88.8	181/209	86.6	13.95	294	0.0001
4	248/275	90.2	164/188	87.2	11.21	242	0.0001
5	253/281	90.0	181/223	84.3	14.93	430	0.0001
6	253/288	87.8	174/209	83.3	9.56	259	0.0001
7	238/269	88.5	183/219	83.6	15.90	355	0.0001
8	249/275	90.5	185/219	84.5	13.72	316	0.0001
9	226/274	82.5	186/215	86.5	17.20	423	0.0001
10	242/260	93.1	194/223	87.0	14.87	324	0.0001

*Mean*		88.5		86.0			

Abbreviations: Num, number of, CON, normal control, ASD, Autism Spectrum Disorder; t, T-test; df, degrees of freedom, *P*, probability value. Results are the number and percent of correctly classified Test Set subjects. T values are determined for each test-set using the corresponding training-set-developed developed discriminant function.

### Age subgroup analyses, two group dfa, 40 coherence factors

#### Ages 2 to 4 years

When, first, all 40 coherence variables were forced to enter DFA on the 2- to -4-year-old population of 301 subjects (C-group, n = 85; ASD-group, n = 216), group differentiation by Wilks' Lambda (0.210), with Rao's approximation (F = 24.50; df = 40,260; *P *< 0.0001) was highly significant. The C-group subjects were classified with 92.9% accuracy, the ASD-group patients with 99.5% accuracy.

Second, when stepping in and out of all 40 variables was allowed, 17 variables were selected with excellent direct classification success for the C- (90.6%) and the ASD- (98.6%) groups. Jackknifing revealed almost identical results: for the C-group, classification success was 90.6%, for the ASD-group, 98.1%.

#### Ages 4 to 6 years

When, first, all 40 variables were forced to enter DFA on this population of 137 subjects (C-group, n = 22; ASD-group, n = 115), despite the unequal subject number per group, a highly significant group differentiation was again observed by Wilks' Lambda (0.155), with Rao's approximation (F = 13.09; df = 40,96; *P *< 0.0001). The C-group subjects and the ASD-group patients were both classified with 100% accuracy.

Second, when stepping in and out of all 40 variables was allowed, 17 variables were selected; direct classification success was excellent for the C-group (90.9%), as well as the ASD-group (99.1%). Jackknifing revealed identical results.

#### Ages 6 to 12 years

When, first, all 40 variables were forced to enter on the population of 546 subjects (C-group, n = 447; ASD-group, n = 99), group differentiation of C- and ASD-group subjects by Wilks' Lambda (0.278), with Rao's approximation (F = 32.80; df = 40,505; *P *< 0.0001) was again highly significant. The C-group subjects were classified with 98.7% and the ASD-group patients with 96.0% accuracy.

Second, when stepping in and out of all 40 variables was allowed, 22 variables were selected with excellent direct classification success (C-group, 98.7%, ASD-group, 96.0%). Jackknifing revealed similar results (C-group, 98.7%, ASD-group, 93.9%).

The highly significant group differentiation results for all three analyses, when all 40 factors were forced to enter, establishes that coherence factors demonstrate significant ASD- and control-group difference across all three age spans. Furthermore, the coherence factors accurately classified ASD- and C-group subjects across all three age spans with jackknifing, when variable stepping in and out was allowed.

### Characteristics of coherence factor differences between ASD- and control-groups

Of the 40 coherence factors, 33 were selected for use in one or more stepwise DFA. Figure 1 shows electrode locations involved and their respective names; Figure 2 illustrates the 33 coherence factors. In Figure 2 lines indicate electrode pairs and the color signifies coherence change relative to the ASD-group; red indicates increased and yellow decreased coherence for the ASD-group as compared to the C-group. Other studies [49,53,57] have utilized the conventionally accepted way to capture the most important coherences per factor, namely by identification of the coherence with the highest loading value per factor and additional display of all other coherence loadings that achieve within 85% or more of the highest loading value on the factor.

The first 10 factors chosen by stepwise DFA and their order of selection are shown in Table 5 columns 3 to 6 respectively for the 2- to 4-year-olds, the 4- to 6-year-olds, the 6- to 12-year-olds, and the entire 2- to 12-year-old sample analyses. Column 7 indicates the number of times each factor was selected over the 10 split half replications of the 2- to 12-year-old population. Column 8 shows the average order of factor selection for the same ten10 replications.

**Table 5:** Factor spectral range and factor utilization across all analyses

		Rank of First 10 Chosen Factors					
				
Factor	Spectral Band Hz (peak)	2 to 4 yo	4 to 6 yo	6 to 12 yo	2 to 12 yo	Split-Half Num 2 to 12 yo	Split-Half Avg Rank 2 to 12 yo
1	12 to 18 (14)	9	8	-	-	9	6.5
2	2 to 20 (18)	2	-	3	3	9	4.2
3	14 to 30 (24)	-	-	-	-	1	7.0
4	16 to 30 (24)	-	-	-	-	0	-
6	14 to 24 (22)	4	-	-	5	8	6.5
7	18 to 30 (22)	-	-	-	-	5	6.8
8	12 to 30 (24)	-	-	-	-	0	-
9	10 to 12 (10)	-	-	8	8	1	8.0
10	6 to 8 (6)	-	-	-	-	0	-
11	22 to 28 (26)	-	-	-	-	0	-
13	4 to 18 (14)	-	5	-	-	0	-
15	12 to 30 (24)	1	-	1	1	10	1.0
16	2 to 4 (2)	-	-	4	4	9	2.6
17	18 to 30 (20)	-	1	6	2	10	3.4
18	4 to 6 (4)	-	-	-	-	0	-
19	16 to 30 (18)	5	-	-	-	0	-
21	14 to 30 (22)	8	3	-	-	0	-
22	18 to 30 (28)	-	-	9	10	2	6.5
23	2 (2)		-	-	-	-	0
24	12 to 28 (18)	3	-	-	6	8	7.7
25	24 to 30 (26)	-	-	5	-	1	10.0
27	8 (8)	-	10	-	-	1	7.0
28	16 to 28 (20)	-	7	-	-	0	-
30	10 to 20 (14)	-	-	10	-	8	7.5
31	16 to 26 (24)	-	-	2	-	3	7.3
32	18 to 22 (18)	-	-	-	-	0	-
33	16 to 28 (20)	-	-	-	-	0	-
34	16 to 26 (20)	-	9	7	-	0	-
35	12 to 24 (14)	-	4	-	8	5	7.25
36	18 to 22 (20)	10	-	-	9	4	7.00
37	12 to 24 (20)	6	2	-	-	0	-
39	4 to 12 (10)	7	6	-	-	0	-
40	4 to 16 (6)	-	-	-	7	3	4.00

**Abbreviations**: Hz, Hertz; yo, year old group analysis; Num, number of utilizations of indicated factor in 10 split-half replications; Avg, average factor rank across 10 replications; -, not utilized.

#### Direction of coherence change for ASD- compared to C-group subjects

Based on coherence loadings upon the 33 factors utilized (Figure 2) and upon the subsequent factor loadings on the individual discriminant, 23 factors (69.7%) were associated with reduced coherence and 10 factors (30.3%) with increased coherence for the ASD population. No single factor manifested a mixture of increased and decreased coherence loadings.

#### Electrode Involvement and Direction of Coherence Change

A tally across the 33 coherence factors (Figure 2) showed frontal electrode involvement in 16, central in 14, occipital in 16, parietal in 16 and temporal involvement in 24 factors. Five frontal, 3 central, 3 parietal, 3 occipital and 10 temporal electrodes were utilized in this study (Figure 1). Thus, the preponderance of temporal electrode involvement in the 33 factors may simply represent the relatively greater number of temporal electrodes utilized.

As regards direction of coherence change by region (Figure 2), increased coherence for the ASD-group was evident in 7 of 16 frontal (43.8%), 3 of 14 central (21.4%), 5 of 16 parietal (31.25%), 3 of 16 occipital (18.8%) and 9 of 24 temporal (37.5%) electrodes. With the exception of the frontal electrodes, these values differ only slightly from the overall 30.3% of the factors that showed increased ASD coherence.

Regionally (Figure 2), 23 of the 33 factors (69.8%) demonstrated bilateral involvement although 2 of these 23 factors illustrated greater left sided involvement. Primarily lateralized involvement was noted on the right for seven (21.2%) and on the left for three (9%) factors.

#### Spectral bands involved

Table 5, column 2, shows the peak frequency and spectral range for each factor. The average spectral range per factor was 10.1 Hz with a range extending from 2-18 Hz. Table 6, last line, columns 2 to 6 shows that based upon peak frequency for each factor there were 2 delta (2 Hz), 4 theta (4 to 8 Hz), 2 alpha (10 to 12 Hz), 17 slow beta (14 to 22 Hz) and 8 fast beta (24 to 30 Hz) factors.

**Table 6:** Relationship of spectral bands to interelectrode distance of factors

Length and Loading	Delta 2 Hz	Theta 4 to 8 Hz	Alpha 10 to 12 Hz	Slow Beta 14 to 22 Hz	Fast Beta 24 to 30 Hz	Totals
Long Pos	2	0	0	5	2	9
Long Neg	0	2	2	6	1	11
						20 Long
						
Mixed Pos	0	0	0	0	0	0
Mixed Neg	0	0	0	2	3	5
						5 Mixed
						
Short Pos	0	0	0	1	0	1
Short Neg	0	2	0	3	2	7
						8 Short
Totals	2	4	2	17	8	

Abbreviations: Pos, positive loading on factor for ASD; Neg, negative loading on factor for ASD; Hz, Hertz

#### Factor inter-electrode distance, loading polarity, and spectral association

Short inter-electrode distance was defined as an adjacent electrode pair without intervening inter-hemispheric fissure; all others were considered long inter-electrode distances. Of the 33 factors utilized, 20 were characterized predominantly by long, five by mixed short and long, and eight by short distance factors (Figure 2, Table 6). The long distance coherence factors were composed almost equally by factors demonstrating positive and negative coherence loadings. The mixed long and short and the short distance coherence factors demonstrated primarily decreased coherences for the ASD group. Nine of the 10 positive loading factors were in the long distance category. Overall, more factors involved the slow beta band than any other band (Tables 5 and 6).

#### Number of coherence loadings per factors and spectral relationship

Eight factors demonstrated loadings limited to a single electrode pair, 11 factor loadings involved 2 or 3 pairs, and 14 factor loadings involved more than 3 pairs (Figure 2, Table 7). There was no obvious relationship between factor coherence electrode distance and/or involved spectral bands (Table 7).

**Table 7:** Relationship of spectral bands to coherences and sign of loading per factor

Num of Coherences Per Factor	Load	Delta 2 Hz	Theta 4 to 8 Hz	Alpha 10 to 12 Hz	Slow Beta 14 to 22 Hz	Fast Beta 24 to 30 Hz	Totals
1	Pos	0	0	0	1	1	2
	Neg	0	0	1	3	2	+6
							8 (1)
							
2-3	Pos	1	0	0	2	1	4
	Neg	0	2	0	5	0	+7
							11 (2 to 3)
							
> 3	Pos	1	0	0	3	0	4
	Neg	0	2	1	3	4	+10
							14 (> 3)

**Abbreviations: **Num, number; Pos, positive loading on factor; Neg, negative loading; Hz, Hertz; See text for definitions of 1, 2-3, and > 3; Load, coherence loading on factor for ASD

#### Most useful factors

Factor 15 was ranked first for every one of the 10 split half analyses of the entire 2- to 12-year-old population. Other factors frequently chosen and/or highly ranked were factors 17, 16 and 2 (Table 5, column 7 and 8). Factor 15 was also chosen first by stepwise DFA for three of the four subgroup analyses (Table 5, columns 3 to 6).

## Discussion

The discussion focuses first on methodological contributions of the current study of children with ASD, and second, on results obtained in view of the study's specific goals.

### Methodological contributions

First, subjects were not selected from among the typically more cooperative population of adult patients with autism, pediatric patients presenting with high-functioning autism or pediatric patients with Asperger's syndrome. Instead, our subjects represented a mid-range cross-section of childhood autism and PDD-nos as referred to area specialists. The EEG technologists who performed the data acquisition were highly experienced in the EEG studies of pediatric patients who frequently require special management in order to acquire useful data. Second, with the anticipation that such patients would none-the-less likely provide data containing some group specific artifact, a special process was employed to recognize, hopefully remove and at least diminish ASD-group specific artifact. Third, an equally large database of well studied inclusive of EEG, neuro-typical children of comparable age and gender distribution was available for comparative purposes. Fourth, instead of *a priori *limitation of EEG coherence to certain scalp channels or spectral frequencies as is frequently the case, all available scalp channels and spectral bands were utilized by employment of a method of data reduction based on Principal Components Analysis (PCA) [52,53], which has previously been used successfully [57]. Fifth, while a number of studies report identified significance of group difference only, the current study took advantage of the large population size and tested stability of individual subject classification. Sixth, evaluation of the coherences loadings upon the most useful PCA-derived factors facilitated, identified not only spectral frequencies (see Figure 2) but also brain regions (see Figure 2) involved in the discrimination of ASD- from control group subject.

### Study goals and findings

The first goal of the study was to determine whether coherence factors, here used as variables, significantly separate ASD- from the control (C)-group populations. As described under Results, when all 40 variables were forced to enter, discriminant function analysis (DFA) produced a highly significant (*P *< 0.0001) group difference across the full 2- to 12-year-old population and, additionally, for the three separate age group analyses of the 2- to 4-, the 4- to 6-, and the 6- to 12-year-old subjects. These findings establish that the 40 coherence factors significantly separate pediatric ASD-patients from C-subjects.

The second goal was to evaluate the consistency of subject classification by allowing DFA to select the best factors for discrimination. As discussed in Results the average jackknifed classification success for the three separate age-group DFAs was 93.7% for the control- and 97.0% for the ASD-group. When the entire population was subjected to 10 independent split half replications, classification success was on the average 88.5% correct for the C- and 86.0% correct for the ASD-groups. Moreover, when each training-set-generated discriminant function was evaluated against the corresponding test-set by t-test, every one of the 10 control- versus ASD-group comparisons reached probability levels of *P *< 0.0001 levels. These findings thus establish coherence factors as very useful in subject classification. They, furthermore, establish the substantial stability of the reported coherence findings and argue quite strongly against great inter-subject variability in this study's ASD population. The illustrated factor coherence loading patterns (Figure 2) appear to constitute a potentially useful neurophysiological ASD-phenotype. Furthermore, the demonstrated stability of the above coherence findings argues against marked variability of brain function within the ASD population as postulated by Happé [32] and Milne [33].

It is tempting to speculate that the consistency of the classification success reported might point to EEG coherence as a possible future diagnostic test for ASD. However, clinical patients are seldom referred just to confirm that they are either neuro-typical or warranting the diagnosis of ASD. Rather, they are referred to establish a diagnosis from among a wide range of clinical possibilities that may produce clinical presentations superficially similar to ASD, including ASD itself. Before entertaining general clinical applicability, the discriminant process will need to be extended to correctly classify conditions beyond the simple C- versus ASD-group dichotomy. Further analyses must encompass diagnoses often associated with or closely related to classic ASD, such as GDD, Asperger's syndrome, developmental dysphasia, childhood disintegrative disorder and autistic behavior as a presenting symptom of other clinical diagnoses, for example, Rett's syndrome, Angelman's syndrome, tuberous sclerosis and Fragile X syndrome.

The controversy of whether childhood disintegrative disorder and especially Asperger's disorder, should or should not be folded into the ASD-category as DSM-V argues [1,14,71], might be answered by similarities and/or differences found on EEG coherence and possibly other neuroimaging tests. Wing *et al. *[71] have argued "We, in our many years of clinical diagnostic work have observed how extremely difficult, even impossible, it is to define boundaries of different sub-groups among children and adults with autistic spectrum conditions." The authors' clinical experience parallels this view.

A third goal of the current study was to explore the potential meaning of the 33 factors chosen (as best to discriminate between ASD- and C-group subjects) by the multiple DFAs when variables were allowed to step in and out.

In studies of EEG coherence, careful pre-selection of electrode pairs has been frequently undertaken prior to data analysis, for example, see Coben *et al. *[23]. This study involved a sample of anterior to posterior intrahemispheric (for example, F3-O1), left to right interhemispheric (for example, C3-C4), and intra-lobar (for example, T7-P7) electrode pairs - see Figure 1 for named electrode locations. Such electrode pair selection facilitates subsequent discussion of coherence increase/decrease in particular frequencies, in different regions, between short and long distance coherence as well as between hemispheres. In contrast, for the current study, channel pairs were not pre-selected; instead exclusively data driven factor loading patterns were used to define coherence pair groupings (Figure 2). As became apparent, none of the factor loading patterns delineated any electrode pairs that reflect simple left-right or anterior-posterior orientations of the sort pre-selected in earlier studies (for example, [23]). On the one hand, this complicates a direct comparison of the current study's findings with prior studies. On the other hand, since the patterns of coherence pair associations in Figure 2 were driven exclusively by the data structure underlying the large study population's coherence data, they may be taken to represent coherence channel pairs that are the most likely to associate with one another in the larger ASD population and, therefore, the most likely to discriminate ASD- from C- subjects. Despite the complexity of patterns identified none-the-less orderly generalizations about coherence difference in ASD emerge from the results.

Overall, 70% of the factors were associated with reduced coherence for the ASD- population. Furthermore, two of the four most utilized factors by DFA, including the most frequently selected Factor 15, were characterized by reduced ASD coherence. Moreover, seven of the eight factors characterized by short inter-electrode distance and all five of the factors representing a mix of short and long distance coherences were associated with reduced coherence. This study is not, of course, the first to report evidence for reduced coherence in ASD [22,23,25,27,28]. Such a preponderance of reduced coherence in ASD suggests likely corresponding reduction in cortical connectivity and corresponding lack of interactions between cortical regions. Some authors attribute ASD primarily to reduced integration of brain activity where specialized cortical regions are anatomically and functionally poorly connected with one another [17,72-76]. Indeed, the most consistently selected factor in the current study (Factor 15) exclusively demonstrated reduced connectivity primarily between the posterior and anterior left temporal regions, and between the left anterior temporal and left frontal regions - and to a degree in the right anterior temporal region. Broadly, left temporal-frontal regions are associated with language function; reduced connectivity in these regions may be associated with the language and communication challenges that are nearly universal in the ASD population. Factor 15 may represent decreased connectivity along the left hemisphere's Arcuate Fasciculus, an anatomical tract important in language and recently shown to be deficient in autism [77].

On the other hand, 30% of the 33 factors utilized in the current report represented increased ASD-coherence. The current study again is not the first to report evidence for increased coherence in concert with reduced coherence [22,27,28] with some studies reporting primarily increased coherence [21,26]. It is more difficult to interpret increased connectivity in the context of ASD-subjects. Increased connectivity, as seen in this study, is primarily represented by long inter-electrode distance factors. This might represent a failure of developmentally appropriate pruning or die-back and, thereby, constitute a further functional liability. Failure of expected die-back of certain cortical-cortical connections with the attendant, aberrant over-connectivity might interfere with normal cortical processing. An alternative possibility is that the increased coherence may constitute a compensatory attempt of the autistic brain to form atypical, spatially disparate, cortical networks in an attempt to replace function normally subserved by assumed-to-be deficient more localized networks. Additionally, the presence of increased coherence might relate to the known association between autism and epilepsy [78].

This study identified no evidence for consistent lateralization among the factor loading patterns and no overriding regional involvement. Furthermore, this study identified no clear inter-relationships among spectral bands, number of coherences per factor, nor increased or decreased coherence. A primary spectral finding was the dominance of slow beta across all conditions with the majority of factors manifesting peak loadings in the slow beta range and far fewer in the fast beta, theta, alpha and delta ranges, a finding of uncertain clinical significance. Earlier studies which demonstrated findings specific to differing scalp regions and spectral ranges may largely reflect methodological differences as discussed in the Background.

The most remarkable spectral finding in the current study was the broad, more than 10 Hz wide, average spectral range per factor, with factor spectral bandwidths ranging up to 18 Hz. In other words, within the ASD population coherence patterns tended to be unusually stable across broad spectral ranges, a finding not reported in previously studied non-ASD populations whose ages ranged from infancy to adulthood [53,57]. The unusually broad spectral ranges in the ASD population, as evidenced for the majority of coherence factors, may reflect yet another characteristic of abnormal neurophysiology in ASD. An understanding of this unexpected finding of unusually broad spectral ranges per factor may be gained by drawing analogies to and making possible inferences from the spectral filtering characteristics of complex systems in electrical and/or mechanical engineering [79]. A spectral filter may be defined as a network or circuit that transmits or passes certain frequencies from its input to its output, its pass band, while rejecting other frequencies. On an input/output plot a narrow or sharp filter has a well defined peak response associated with a rapid fall-off on either side, that is, a narrow pass-band. A wide or broad filter, in contrast, possesses a wide pass-band with slow roll-off on either side of a less distinct peak. The "Q" of a filter is a dimensionless number that characterizes a resonant circuit's bandwidth relative to its center frequency. This feature also serves as an indication of how damped a circuit may be. As a physical example of a high Q filter, one might consider a thin, high quality crystal goblet. As an example of a low Q physical filter, one might consider a typical, ceramic coffee mug. High Q circuits are relatively easy to activate, for example, tapping the crystal goblet causes a sustained ringing of moderate amplitude at a single frequency reflecting its narrow pass band and sharp resonance peak, whereas low Q circuits, for example, tapping the ceramic coffee mug produce a brief, low amplitude, broad frequency "thunk" at best. Thus, low Q circuits are more damped than high Q circuits [79].

Returning to the broad frequency bands identified in the current study, the complex coherence patterns outlined by the factor loadings may serve to identify important 'damped' processing circuit characteristics within the ASD-brain. Factor 15 may reflect reduced connectivity in an important cortical auditory processing circuit. Although it peaks at 24 Hz, there is very little change in Factor 15 loading patterns across a wide pass band from 12-30 Hz - the pattern of a putative low Q, wide bandwidth, heavily damped system. It may be unusually difficult for this circuit to be driven into action by external stimulation, such as speech input. One might speculate that the typical lack of response to verbal input in autism may reflect not the absence of needed cortical circuitry but a poorly responding, low Q circuit response of language cortex that is postulated to be overly damped. The autistic auditory cortex may act more like the coffee mug than the crystal goblet. One might further speculate that there may be intrinsic biological factors in the autistic brain that dampen, inhibit or otherwise limit responsiveness in general, given the overall wide spectral ranges and predominant decrease of connectivity that characterize the coherence factor loading patterns.

## Conclusions

Extensive spectral coherence data sets may be reduced by PCA to a much smaller number of factors accounting for a large fraction of underlying variance. Such factors, when treated as variables, significantly separate C-group from ASD-group children by DFA. Moreover, DFA-derived discriminant functions reliably classify individual control-group and ASD-group subjects prospectively as demonstrated by jackknifing and repetitive split half replication.

The demonstrated classification stability across replications suggests that the coherence loading patterns might constitute a first prototype for an EEG-coherence-based neurophysiological phenotype of ASD.

There appears to be a preponderance of diminished coherence in ASD patients as others have also reported. The most utilized factor in DFA, namely Factor 15, primarily represents reduced coherence in the left temporal-frontal regions possibly reflecting altered connectivity in the Arcuate Fasciculus. It is likely related to diminished language dysfunction in ASD-patients. The slow beta spectral band was the most actively involved, yet the primary spectral finding was that of a very wide frequency spread that was associated with most factors. It is speculated that this may represent evidence for overly damped but otherwise intact ASD cortical circuitry, which could explain the delayed, incomplete responsivity that often characterizes ASD-patients behaviorally.

It is speculated that spectral coherence data may prove useful in exploration of similarities and differences within a broader population of autistic children and adults. Spectral coherence alone may also assist in the early detection of ASD in younger children including infants and/or it might be helpful in concert with additional techniques of EEG analysis such as "complexity" measures [80] among others.

## Abbreviations

ADD: attention deficit disorder;ADHD: attention deficit hyperactivity disorder; ADOS: Autism Diagnostic Observation Schedule; ASD: autism spectrum disorder; C: control; CHB: Children's Hospital Boston; CSD: current source density; df: degrees of freedom; DFA: discriminant function analysis; DMRI: diffusion MRI; DSM: Diagnostic and Statistical Manual; EEG: electroencephalogram: electroencephalography; EP: evoked potential; FA: functional anisotropy (MRI); FFT: Fast Fourier Transform; fMRI: functional MRI; GDD: global developmental delay; IRB: Institutional Review Board (CHB); IVH: intraventricular hemorrhage; MRI: magnetic resonance imaging; PCA: principal components analysis; PDD-nos: pervasive developmental disorder not otherwise specified; Q: a dimensionless number that characterizes a resonant circuit's bandwidth relative to its center frequency (electrical engineering); SVD: singular value decomposition

## Competing interests

The authors declare that they have no competing interests.

## Authors' contributions

FHD and HA contributed to the study's concept and design, selection of patients and subjects, and interpretation of results. FHD contributed to acquisition and preparation of neurophysiologic data and statistical analyses. FHD had full access to all the data in the study and takes responsibility for all aspects of the study, including integrity of the data accuracy and the data analysis. Both authors collaborated in writing and editing the paper and approved the final manuscript.

## Authors' information

FHD is a physician, child neurologist, clinical electroencephalographer and neurophysiologist with degrees in electrical engineering and mathematics. Current research interests are in neuro-developmental disorders and epilepsy, including the development and utilization of specialized analytic techniques to support related investigations. HA is a psychologist with research interests in newborn, infant and child neuro-development, including generation of early predictors of later outcome from behavioral, MRI and neurophysiological data.

## Pre-publication history

The pre-publication history for this paper can be accessed here:

http://www.biomedcentral.com/1741-7015/10/64/prepub
